# Abelson Helper Integration Site 1 haplotypes and peripheral blood expression associates with lithium response and immunomodulation in bipolar patients

**DOI:** 10.1007/s00213-023-06505-5

**Published:** 2023-12-01

**Authors:** Kosma Sakrajda, Karolina Bilska, Piotr M. Czerski, Beata Narożna, Monika Dmitrzak-Węglarz, Stefanie Heilmann-Heimbach, Felix F. Brockschmidt, Stefan Herms, Markus M. Nöthen, Sven Cichon, Barbara Więckowska, Janusz K. Rybakowski, Joanna Pawlak, Aleksandra Szczepankiewicz

**Affiliations:** 1 Molecular and Cell Biology Unit, Poznan University of Medical Sciences, Poznan, Poland; 2 Doctoral School, Poznan University of Medical Sciences, Poznan, Poland; 3 Department of Psychiatric Genetics, Poznan University of Medical Sciences, Poznan, Poland; 4 Institute of Human Genetics, University of Bonn, Bonn, Germany; 5 Department of Genomics, Life & Brain Center, University of Bonn, Bonn, Germany; 6 Department of Biomedicine, University Hospital Basel and University of Basel, Basel, Switzerland; 7 Institute of Medical Genetics and Pathology, University Hospital Basel, Basel, Switzerland; 8 Institute of Neuroscience and Medicine (INM-1), Research Centre Jülich, Jülich, Germany; 9 Department of Computer Sciences and Statistics, Poznan University of Medical Sciences, Poznan, Poland; 10 Department of Adult Psychiatry, Poznan University of Medical Sciences, Poznan, Poland

**Keywords:** Bipolar disorder, Lithium, Abelson Helper Integration Site 1 (*AHI1)*, Immunomodulation

## Abstract

**Rationale:**

In bipolar disorder (BD), immunological factors play a role in the pathogenesis and treatment of the illness. Studies showed the potential link between Abelson Helper Integration Site 1 (AHI1) protein, behavioural changes and innate immunity regulation. An immunomodulatory effect was suggested for lithium, a mood stabilizer used in BD treatment.

**Objectives:**

We hypothesized that AHI1 may be an important mediator of lithium treatment response. Our study aimed to investigate whether the *AHI1* haplotypes and expression associates with lithium treatment response in BD patients. We also examined whether *AHI1* expression and lithium treatment correlate with innate inflammatory response genes.

**Results:**

We genotyped seven *AHI1* single nucleotide polymorphisms in 97 euthymic BD patients and found that TG haplotype (rs7739635, rs9494332) was significantly associated with lithium response. We also showed significantly increased *AHI1* expression in the blood of lithium responders compared to non-responders and BD patients compared to healthy controls (HC). We analyzed the expression of genes involved in the innate immune response and inflammatory response regulation (*TLR4*, *CASP4*, *CASP5*, *NLRP3*, *IL1A*, *IL1B*, *IL6*, *IL10*, *IL18)* in 21 lithium-treated BD patients, 20 BD patients treated with other mood stabilizer and 19 HC. We found significantly altered expression between BD patients and HC, but not between BD patients treated with different mood stabilizers.

**Conclusions:**

Our study suggests the involvement of *AHI1* in the lithium mode of action. Moreover, mood-stabilizing treatment associated with the innate immunity-related gene expression in BD patients and only the lithium-treated BD patients showed significantly elevated expression of anti-inflammatory *IL10,* suggesting lithium’s immunomodulatory potential*.*

## Introduction

Bipolar disorder (BD) is a lifelong psychiatric disorder characterized by recurrent, episodic fluctuations of mood and energy affecting over 1% of the population worldwide (Vieta et al. 2018; Carvalho et al. 2020). The previous studies underlined the role of inflammatory disturbances and the immune response in the central nervous system and periphery as the potential pathophysiology underlying mood disorders (including BD). These changes in the immune system may lead to the increased risk of mood disorders and poor response to antidepressants and mood stabilizers, thus influencing the course of disease (Rosenblat and McIntyre 2016; Fries et al. 2019; Sakrajda and Szczepankiewicz 2021). The immunomodulatory potential of some drugs (including lithium) seems like a promising solution overcoming these therapeutical difficulties.

Lithium has been an efficient mood stabilizer used in the pharmacotherapy of psychiatric disorders for over 70 years (Cade 1949; Won and Kim 2017). The primary mechanism of action is the direct inhibition of glycogen synthase kinase-3β (GSK-3β) and phosphatidylinositol pathway (Won and Kim 2017). However, recent studies have shown that lithium affects many other processes, including antiviral response and immunomodulatory potential related to the effectiveness of pharmacotherapy (Nassar and Azab 2014; Dong et al. 2014; Li et al. 2018; Adams et al. 2020; Queissner et al. 2021; Landén et al. 2021; Le Clerc et al. 2021; Sakrajda et al. 2022; Rybakowski 2022). The study by Lancaster et al. (2011) also showed the link between lithium and Abelson Helper Integration Site-1 (AHI1) function. Authors presented that lithium treatment of pregnant *Ahi1* knock-out dams with Joubert syndrome partially restored the normal phenotype (intact midline fusion and increase in the number of proliferating cells) in *Ahi1* knock-out embryos compared to the animals not treated with lithium.

*AHI1* encodes the Jouberin protein required for ciliogenesis and brain neurodevelopment. Its malfunction underlies Joubert syndrome and it may also be involved in the pathogenesis of neuropsychiatric disorders including schizotypal disorder or Alzheimer’s disease (Dixon-Salazar et al. 2004; Lancaster et al. 2011; Schmitz et al. 2019; Sheu et al. 2020). The *AHI1* gene is located on the long arm of chromosome 6q23, a region associated with susceptibility to mood disorders (Zubenko et al. 2004). The *AHI1* genetic variability was also previously associated with stress response, schizophrenia (SCZ) and mood disorders (Amann-Zalcenstein et al. 2006; Rivero et al. 2010; Ingason et al. 2010). Functional studies showed that *AHI1* knock-outs induced the depressive-like behaviour in the animal models (Ren et al. 2014). Interestingly, a recent study reported that AHI1 also regulates the innate immune response in major depressive disorder (MDD) patients and depressive-like mice (Zhang et al. 2022). So far, it was not studied if *AHI1* may be associated with lithium response in bipolar patients and if its expression correlates with innate immunity changes in bipolar disorder.

Therefore, our study aimed to investigate whether the *AHI1* haplotypes and expression associates with response to lithium treatment in BD patients. We also aimed to investigate the relationship between AHI1, lithium treatment and innate inflammatory response gene expression.

## Experimental procedures

### Samples

Our study included 97 patients with bipolar disorder treated in the Department of Adult Psychiatry Poznan University of Medical Sciences (Poznan, Poland). The diagnosis was performed using the structured clinical interview for DSM-IV (SCID). The patients were treated with lithium carbonate for at least 5 years. The treatment response was assessed using the retrospective assessment of the lithium response phenotype scale (Alda scale) and the score ≥ 7 was considered as the response to lithium treatment (Nunes et al. 2020). Samples for gene expression analysis included 21 BD lithium-treated patients, including 6 lithium responders (Alda ≥ 7), 6 lithium non-responders (Alda ≤ 3) and 9 partial-responders (Alda 4–6). We also included the group of BD patients (*n* = 20) treated with mood-stabilizing drugs other than lithium (valproic acid, carbamazepine, lamotrigine) as well as the healthy control group (*n* = 19). Both BD patient groups included in the analysis were collected in an euthymic state, defined as the reduction in symptom manifestation (less than 6 points on the Hamilton Depression rating Scale or less than 6 points on the Young Mania Rating Scale). The lithium-treated patients received lithium carbonate in doses ranging from 250 to 1000 mg/day, depending on the clinical state of the patient based on psychiatrist assessment and decision. The lithium serum concentration was measured regularly and was maintained in the therapeutic range of 0.4–1 mmol/L. The samples were collected as a naturalistic sample without intervention in the treatment protocol applied in the Department of Adult Psychiatry at Poznan University of Medical Sciences (Poznan, Poland). The study was approved by the local Bioethics Committee (agreements number 28/08, 1194/16 and 758/17).

### *AHI1* genotyping and lithium response assessment

DNA genotyping included blood samples from 49 non-responding lithium-treated BD patients (Alda ≤ 3) and 48 lithium-responding BD patients (Alda ≥ 7) (Table 1). We performed the genotyping of seven *AHI1* single nucleotide polymorphisms (SNPs): rs9321501, rs11154801, rs7750586, rs9647635, rs7739635, rs9494332 and rs1475069 using iPLEX assay on the MassARRAY MALDI-TOF mass spectrometer (SEQUENOM, San Diego, CA, USA) as described previously (Ingason et al. 2010). The genotyped SNPs were chosen based on the previous studies associating them with psychiatric disorders (Amann-Zalcenstein et al. 2006; Ingason et al. 2007, 2010). To predict potential functionality of analyzed SNPs, we used the SNPinfo tool (Xu and Taylor 2009).

**Table 1:** Patients with bipolar disorder involved in DNA genotyping analysis

Characteristic	Lithium non-responders	Lithium responders	*p* value
*n*	49	48	
Mean age (± SD)	50 (± 11)	53 (± 11)	0.18
Mean age at onset (± SD)	30 (± 10)	30 (± 10)	0.42
Sex (females/males)	29/20	31/17	0.58
Bipolar disorder (type 1/type2)	13/8	11/9	0.97

### Gene expression analysis

Gene expression profile included samples from 21 BD lithium–treated patients, 20 BD patients treated with a mood stabilizer other than lithium and 19 healthy volunteers (Table 2). Gene expression profile of Toll-like receptor 4 (*TLR4*), NLR family pyrin domain containing 3 (*NLRP3*), caspase 4 (*CASP4*), caspase 5 (*CASP5*), interleukin-1 beta (*IL1B*), interleukin-1 alpha (*IL1A*), interleukin-6 (*IL6*), interleukin-10 (*IL10*) and interleukin-18 (*IL18*) was performed using SurePrint G3 Human Gene Expression Whole Transcriptome Microarrays (Agilent, Santa Clara, CA, USA). The genes were chosen based on previous reports describing them as involved in inflammasome complex and innate inflammatory response or immune response regulation (Saraiva and O’Garra 2010; Kanneganti 2015; Jones and Jenkins 2018; Van Den Eeckhout et al. 2021). The blood samples from BD patients were collected in the euthymia state.

**Table 2:** Patients involved in gene expression analysis

Characteristic	Lithium treated BD	Non-lithium treated BD	Healthy control	*p* value
*n*	21	20	19	
Mean age (± SD)	46 (± 11)	46 (± 16)	41 (± 11)	0.49
Mean age at onset (± SD)	31 (± 10)	26 (± 10)	N/A	0.42
Sex (females/males)	17/4	19/1	15/4	0.30
Bipolar disorder (type 1/type2)	13/8	11/9	N/A	0.65

### Statistical analysis

The statistical analysis of clinical characteristics of studied groups was performed using *t* test, ANOVA or the Pearson chi-square test depending on the type of data, normality testing and the numbers of compared groups. The genotyping and haplotype analysis results were analyzed using the chi-square test and 1000 permutation after the Hardy–Weinberg equilibrium analysis was performed in Haploview 4.4. The differences in the normalized expression were analyzed using the *t* test or Kruskal–Wallis *H* ANOVA test with Dunn test for multiple comparisons with Bonferroni adjustment depending on the number of compared groups. To check the normality of data distribution, we used the Shapiro–Wilk test, and to check the variance homogeneity, we used Levene’s test using Statistica 13.3 software (TIBCO Software Inc., Palo Alto, CA, USA).

## Results

### Association analysis of *AHI1* genotypes with lithium response

**Fig. 1:**
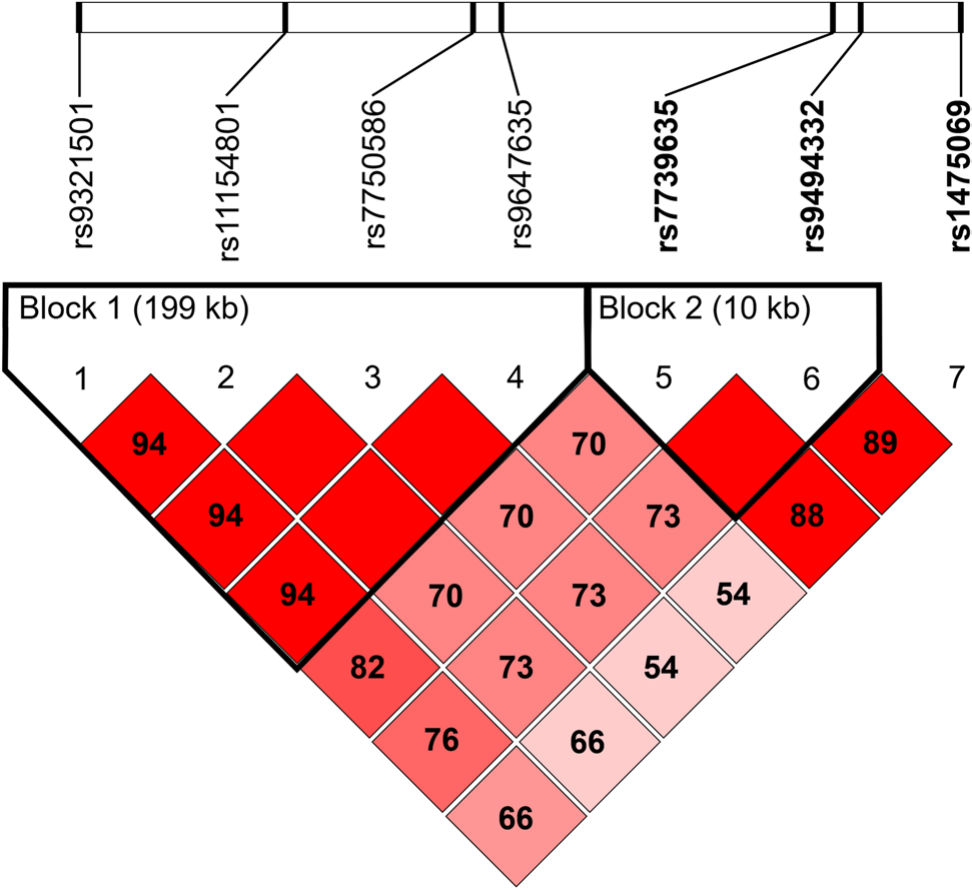
Linkage disequilibrium map of *AHI1* single nucleotide polymorphisms. Three single nucleotide polymorphisms (SNPs) in Abelson helper integration site 1 (*AHI1*) were significantly associated with lithium treatment response (bolded). Two haplotype blocks (bolded frames) were identified within the seven SNPs in *AHI1*. The length of each haplotype block is provided in kilobases (kB), and pairwise linkage disequilibrium (D’) is given for each SNP combination. Empty squares indicate D’ = 1.0. A linkage disequilibrium map of these haplotype blocks was generated using Haploview 4.4

Genotype distribution for all analyzed SNPs was in concordance with the Hardy–Weinberg equilibrium law (*p* > 0.05). The single SNP association analysis showed three *AHI1* polymorphisms: C-rs7739635 (*p* = 0.018), A-rs9494332 (*p* = 0.009) and A-rs1475069 (*p* = 0.025) significantly associated with the lithium response of BD patients assessed with the Alda scale. We identified two haplotype blocks from which one block (block 2) was significantly associated with lithium response: CA (C-rs7739635, A-rs9494332; chi-square = 5.623, *p* = 0.018) and TG (T-rs7739635, G-rs9494332; chi-square = 6.853, *p* = 0.009). The 1000 permutation tests revealed haplotype TG as significantly associated with lithium response (permutation *p* = 0.280) (Fig. 1 and Table 3).

**Table 3:** Haplotypes analysis of *AHI1* polymorphisms with lithium response

Haplotype	Frequency	Non-responders, responders ratios	*p* value	adjusted* p* value
Block 1				
TGTA	0.627	0.664, 0.591	0.292	0.883
GTCC	0.309	0.291, 0.326	0.599	0.986
GGTA	0.053	0.033, 0.072	0.231	0.792
TTCC	0.011	0.011, 0.011	0.988	1.000
Block 2				
CA	0.680	0.760, 0.601	**0.018**	0.055
TG	0.284	0.198, 0.367	**0.009**	**0.028**
TA	0.037	0.042, 0.032	0.697	0.998

Bolded values indicate *p* < 0.05; adjusted *p* value—after 1000 permutations

### Comparative analysis of *AHI1* expression with lithium response

**Fig. 2:**
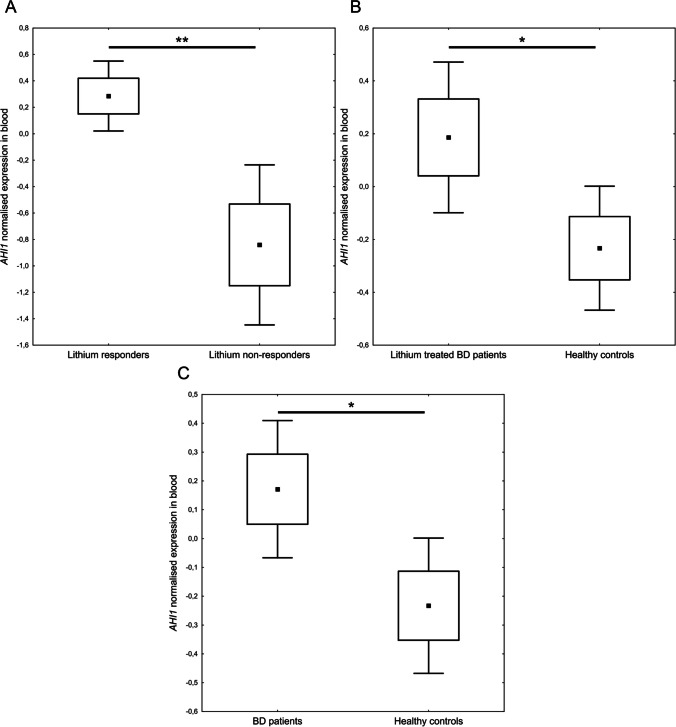
Comparison of *AHI1* expression in blood between lithium responders and non-responders (**A**); lithium-treated BD patients compared to healthy controls (**B**); BD patients regardless of treatment compared to healthy controls (**C**); *p ≤ 0.05, **p ≤ 0.01, black square (▪) indicates mean, boxes indicates mean ± SEM, whiskers indicates mean ± 95% CI

We found that the *AHI1* gene expression was significantly increased in the blood of lithium responders (Alda ≥ 7) compared to BD patients non-responding to lithium (Alda ≤ 3) (*t* = 3.340, *p* = 0.0075) (Fig. 2A). In this analysis, we did not involve the patients showing a partial response to lithium (Alda 4–6) in order to observe more clearly the *AHI1* expression changes characteristic to lithium-responding patients compared to non-responding patients.

Further, we analyzed if the *AHI1* expression differed between lithium-treated BD patients in the euthymic state and the healthy control group, and we found significantly increased *AHI1* expression in lithium-treated BD patients compared to the healthy control group (*t* = 2.197, *p* = 0.034) (Fig. 2B). We also analyzed the *AHI1* expression between the BD patients group (regardless of treatment) and the healthy control group. We found significantly increased *AHI1* expression in the whole BD patients group compared to the healthy control group (*t* = 2.057, *p* = 0.044) (Fig. 2C).

### Innate inflammatory response–related gene expression

Based on the previous reports about the interactions between *AHI1* and the innate inflammatory response (Zhang et al. 2022), we compared the expression of 9 genes related to innate inflammatory response and inflammatory response regulation: *TLR4*, *CASP4*, *CASP5*, *NLRP3*, *IL1A*, *IL1B*, *IL6*, *IL10* and *IL18* between lithium-responders and non-responders. Our analysis did not show significant differences in the expression of any of the genes between these groups of BD patients (Table 4).

**Table 4:** Innate inflammatory response–related gene expression analysis between lithium responders and non-responders

Gene	Mean (± SD) Li-R	Mean (± SD) Li-N	*t* value	*p* value
TLR4	0.468 (± 0.83)	− 0.191 (± 0.49)	1.68	0.11
NLRP3	0.334 (± 0.92)	0.237 (± 0.54)	0.23	0.82
CAPS4	0.412 (± 1.33)	− 0.078 (± 0.52)	0.84	0.42
CASP5	0.559 (± 1.34)	− 0.011 (± 0.55)	0.98	0.35
IL1A	0.385 (± 0.62)	− 0.195 (± 0.60)	1.63	0.13
IL1B	1.067 (± 2.51)	− 0.034 (± 1.15)	0.97	0.35
IL6	− 0.245 (± 1.61)	0.296 (± 1.30)	− 0.64	0.54
IL10	0.304 (± 0.77)	0.173 (± 0.87)	0.28	0.77
IL18	0.557 (± 0.90)	0.0003 (± 0.69)	1.21	0.26

*Li-R* lithium responders, *Li-N* lithium non-responders

**Fig. 3:**
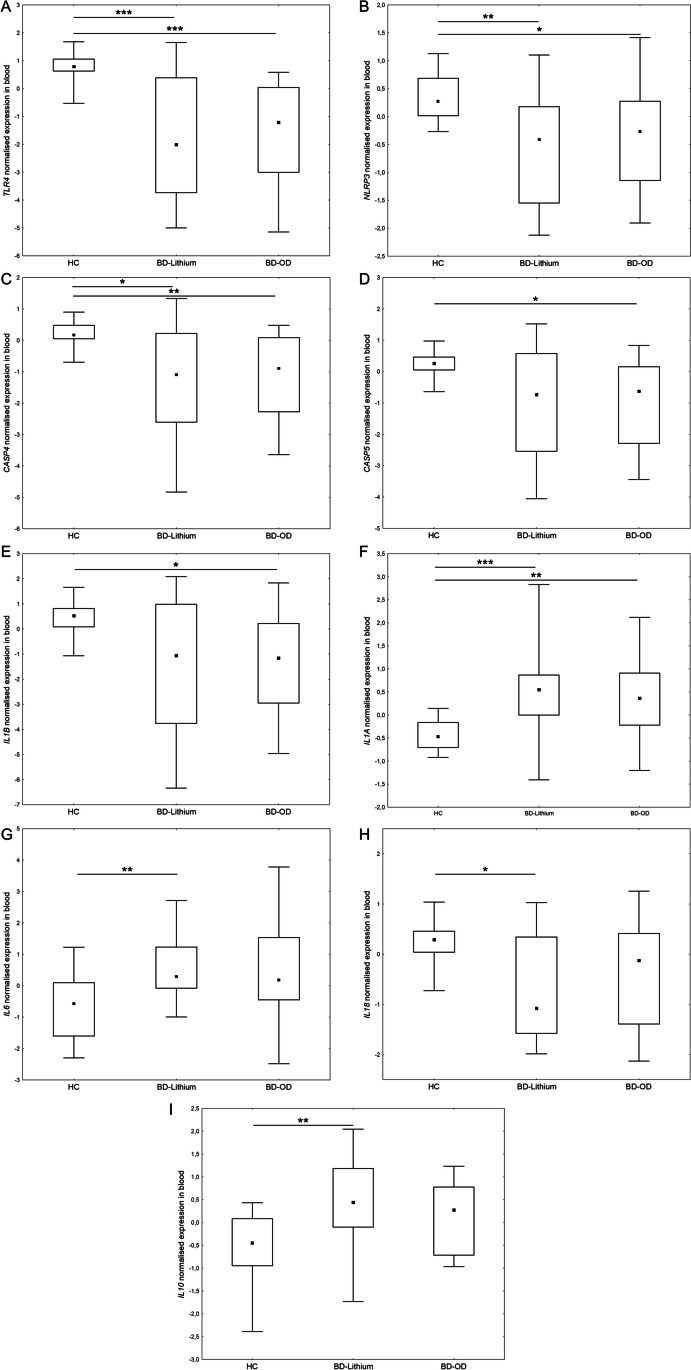
The comparison of expression of innate inflammatory response genes between BD patients treated with lithium (BD-Lithium), BD patients treated with other than lithium mood stabilizer (BD-OD) and healthy controls (HC): Toll-like receptor 4 (*TLR4*) (**A**); NLR family pyrin domain containing 3 (*NLRP3*) (**B**); caspase 4 (*CASP4*) (**C**); caspase 5 (*CASP5*) (**D**); interleukin-1 beta (*IL1B*) (**E**); interleukin-1 alpha (*IL1A*) (**F**); interleukin-6 (*IL6*) (**G**); interleukin-18 (*IL18*) (**H**); interleukin-10 (*IL10*) (**I**); *p ≤ 0.05, **p ≤ 0.01, ***p ≤ 0.001, black square (▪) indicates the median, boxes indicates the 25th and 75th percentile, whiskers indicates minimum and maximum values

To investigate if the changes in the expression of innate immunity genes may be related to bipolar disorder rather than treatment with mood stabilizer, we compared the group of BD patients treated with mood stabilizer other than lithium (BD-OD) with the lithium-treated patients (BD-Lithium) and the healthy control group. We found that the gene expression of these 9 genes differed significantly between BD patients and the control group, but did not differ between BD patients on different mood stabilizers (lithium versus other normotymic agent) (Fig. 3). In BD-Lithium and BD-OD groups, when compared to the control group, we observed a significantly lower expression of *TLR4* (*p* = 0.0002 and *p* < 0.0001, respectively), *NLRP3* (*p* = 0.002 and *p* = 0.014, respectively), *CASP4* (*p* = 0.015 and *p* = 0.005, respectively) and significantly higher expression of *IL1A* (*p* < 0.001 and *p* = 0.002, respectively). We observed significantly lower expression of *CASP5* (*p* = 0.015) and *IL1B* (*p* = 0.028) in the group of BD-OD compared to the control group, but no significant changes in the expression of these genes were observed between the BD-Lithium group and the control group. We also found a significantly increased expression of *IL6* (*p* = 0.007) and *IL10* (*p* = 0.003) and decreased *IL18* expression (*p* = 0.029) in the BD-Lithium group compared to the control group, whereas no significant changes in the expression of these genes were found between the BD-OD and the control group. We did not find significant differences in the innate immunity gene expression between BD-Lithium and BD-OD patients.

## Discussion

The main finding of the study is an association between *AHI1* haplotypes and lithium response and its significantly higher expression in lithium responders than in lithium non-responding BD patients. The expression analysis of innate immunity–related genes did not show any significant differences regarding lithium response in the studied group of lithium-treated BD patients. We also did not find significant differences in innate immunity–related gene expression between BD patients treated with lithium when compared to the BD patients treated with mood stabilizer other than lithium. However, we found differences in immunity-related gene expression when comparing BD patients treated with lithium or BD patients receiving mood stabilizer other than lithium to the healthy controls.

Previous studies reported the relationship between *AHI1* polymorphism and psychiatric disorders, including schizophrenia, autism spectrum, major depressive disorder and bipolar disorder (Alvarez Retuerto et al. 2008; Ingason et al. 2010; Torri et al. 2010; Porcelli et al. 2014; Ren et al. 2016). In our study, we included SNPs that previously showed association with SCZ, BD and MDD, and we found that three SNPs that previously showed association with SCZ (Ingason et al. 2010), rs7739635, rs9494332 and rs1475069, were associated with lithium response in our group of BD patients. The association of these SNPs with SCZ and our study showing their association with the lithium-treatment response in BD may support the common genetic background shared between those disorders (Corponi et al. 2019; Prata et al. 2019).

Our study, for the first time, described an association of *AHI1* haplotypes with lithium response. The haplotype analysis showed that TG haplotype (T-rs7739635, G-rs9494332) is significantly more frequent in lithium responders. Both SNPs are located within introns, and we used the SNPinfo tool to predict the potential functionality of these SNPs. We found that the rs9494332 presents a higher regulatory potential score than rs7739635 (0.136 and 0.000, respectively) and a lower conservation score than rs7739635 (0.001 and 0.798, respectively). However, the functional analysis of these SNPs should be performed to investigate how presented *AHI1* haplotype may influence lithium response.

The in vivo studies indicated the role of AHI1 in mood disorders, showing that the knock-out of Ahi1 resulted in depressive-like behaviour and neurochemical changes in depression-related serotonin and dopamine levels in different brain regions (Ren et al. 2014; Wang et al. 2022). Moreover, the study on a mouse model of Joubert syndrome using Ahi1 knock-out showed that lithium treatment of pregnant dams resulted in a partially restored phenotype in knock-out embryos (Ren et al. 2014). In our study, we observed higher *AHI1* expression in lithium responders compared to non-responders as well as in the lithium-treated BD patients compared to healthy control, which may suggest an association between *AHI1* expression and lithium mode of action. Our study showed also higher *AHI1* expression in the group of euthymic BD patients (regardless of used mood stabilizer) compared to healthy control, which may suggest the role of *AHI1* expression in the course of bipolar disorder.

Studies investigating the immunomodulatory role of AHI1 suggested a link between *AHI1* expression and antiviral immune response in major depressive disorder (MDD) patients (Zhang et al. 2022). The authors showed that the disruption of antiviral innate immune signalling depended on the AHI1-Tyk2 axis in IFN-I cell signalling in drug-free MDD patients and depressive-like mice. They also showed that the decreased *AHI1* expression downregulated Tyk2 and IFN-I signalling. Considering immunomodulatory potential of lithium (Murru et al. 2020; Queissner et al. 2021; Rybakowski 2022), our results suggested that higher *AHI1* expression in peripheral blood influenced lithium response of BD patients. So far, the direct link between lithium response in BD patients and *AHI1* peripheral expression was not studied.

Moreover, to further investigate immunomodulatory potential of lithium, we analyzed the expression of the genes related to innate immune response as possible targets of lithium effect, but we found no significant differences between lithium responders and non-responders Therefore, to further analyze if changes in the expression of innate immune response genes may depend on the disease itself rather than the applied treatment, we compared the expression of these genes between the groups of BD patients treated with lithium with BD patients treated with other mood stabilizer and the healthy control group.

We observed a significant decrease in peripheral expression of several genes: *TLR4*, *CASP4* and *NLRP3* in both BD-Lithium and BD-OD groups compared to the control group. TLR4 is a crucial component of the innate immune system responsible for activating pro-inflammatory cytokines and chemokines expression (Vaure and Liu 2014). The previous study reported the association of the TLR4 genotype with bipolar disorder (Oliveira et al. 2014). The study by Hung et al. (2016) showed decreased *TLR4* expression in the peripheral blood mononuclear cells (PMBCs) of major depressive disorder patients treated with antidepressants as compared to the healthy controls, which is consistent with our results for the BD patients group treated with any mood stabilizer (lithium or other). Similar results were presented in the study by Wieck et al. (2016), who compared the induction of inflammatory response in PBMCs derived from BD patients compared to the PBMCs from healthy controls. The authors also showed that pharmacotherapy decreased the percentage of TLR4( +) monocytes after in vitro stimulation. Interestingly, the study on rats with LPS-induced neuroinflammation showed decreased TLR4 expression after lithium treatment (Khan et al. 2017). Moreover, lithium pre-treatment of LPS-induced cells attenuated TLR4 expression in vitro (Dong et al. 2014; Lu et al. 2015; Li et al. 2016).

In regard to *CASP4/5*, a mediator of non-canonical inflammasome activation via NLRP3 (Mazgaeen and Gurung 2020), our results showed a significant decrease of *CASP4* peripheral expression in both groups of BD patients (BD-Lithium and BD-OD) compared to healthy controls. The study by de Baumont et al. (2015) showed increased expression of *CASP4*, as well as other inflammatory-related genes, in the brains of BD patients compared to SCZ patients and non-psychiatric control brains. Therefore, we suggest that the decreased expression of *TLR4* and *CASP4/5* in both groups of BD patients may be associated with the protective role of mood-stabilizing treatment against inflammasome activation and further inflammatory response.

Based on the results of *TLR4* and *CASP4/5* expression analysis, we further analyzed the expression of *NLRP3* and showed significantly decreased expression in the BD-Lithium (*p* = 0.002) and BD-OD (*p* = 0.01) groups compared to the healthy control group. The NLRP3 protein acts as a sensor of cellular damage and activates the inflammasome complex, a crucial component of the innate and adaptive immune systems. Its activation triggers the release of the pro-inflammatory cytokines such as IL1B and IL18, thus leading to cell death (Elliott and Sutterwala 2015; He et al. 2016; Wang and Hauenstein 2020; Zhang et al. 2021). The NLRP3 activation was previously described in MDD and BD patients (Alcocer-Gómez et al. 2014, 2017; Kim et al. 2016; Scaini et al. 2018; Taene et al. 2020), as well as in the animal models of depression (Pan et al. 2014; Wong et al. 2016; Sahin Ozkartal et al. 2019). These studies showed an increased expression of *NLRP3* resulting in enhanced inflammasome activation and increased levels of IL1B and IL18 cytokines. Both cytokines are NLRP3-dependent cytokines that stimulate inflammatory response. Our results showed that both groups of mood stabilizer–treated BD patients had significantly decreased *NLRP3* expression compared to healthy controls. We also observed changes in *IL1B* and *IL18* expression. However, the changes in these genes were treatment-dependent: *IL1B* expression decreased in the BD-OD group, and *IL18* expression decreased in the BD-Lithium group as compared to healthy controls. These results are consistent with the recent report by Zhao et al. (2022), who showed the lithium-dependent reduction of NLRP3-activated inflammation in the animal model of a spinal cord injury. Similarly, the olanzapine treatment of depressive-like rats showed reduced expression of NLRP3, IL1b and IL18 in serum and hippocampus after treatment as compared to non-treated depressive-like rats (Yue et al. 2019). The reduced IL1B level in BD patients was further confirmed by Chou et al. (2016) who compared peripheral cytokine levels in euthymic BD patients receiving valproic acid with matched healthy controls. Interestingly, the study by Knijff et al. (2007) showed that *IL1B* expression in monocytes from non-lithium-treated BD patients stimulated by LPS was abnormally low but increased after lithium treatment.. In addition, the in vitro study by Himmerich et al. (2014) showed that all analyzed anti-epileptic and mood-stabilizing drugs except lithium reduced IL1B levels in the CD3/5C3 cells and stimulated whole blood samples from healthy volunteers. Therefore, in the context of studies showing the reduced IL1B levels in BD patients, the lack of differences in *IL1B* expression in the BD-Lithium group compared to controls may indicate a restored expression level after the lithium treatment, similar to that observed in healthy subjects.

We also found significant upregulation of *IL1A* expression in BD-Lithium and BD-OD groups compared to healthy controls. The IL1A, a part of the IL1 family of cytokines, is a pro-inflammatory cytokine; however, in contrast to IL1B, the exact mechanism of IL1A signalling is still unknown. *IL1A* is translated as biologically active pro-IL1A, and the cytosolic role of pro-IL1A seems to be bidirectional. On the one hand, it may act as an inflammatory-triggering cytokine in response to necrotic cells signalling involving the IL1R1 receptor and leading to inflammation. On the other hand, the pro-IL1A interaction with inhibitory IL1R2 receptor may reduce or even prevent inflammation (Chen et al. 2007; Zheng et al. 2013; Di Paolo and Shayakhmetov 2016). Although the IL1A release may be controlled by the NLRP3 inflammasome, it may also be released in an NLRP3-independent manner based on cation channels (Groß et al. 2012). Interestingly, the authors presented that an alternative way of IL1A secretion did not affect the inflammasome-dependent IL1B secretion. Our results presented the upregulation of *IL1A* expression and downregulation of *NLRP3* in both groups of BD patients (BD-Lithium and BD-OD) compared to controls, thus suggesting that BD treatment impacts the NLRP3 inflammasome expression but may not affect the alternative way of IL1A expression. Therefore, the increased *IL1A* expression in both groups of BD patients might be the effect of the disorder course, rather than pharmacological intervention. However, these hypothesis needs to be further verified in the functional studies on the role of IL1A in the immune system of BD patients.

Another analyzed cytokine that showed significantly increased expression between BD-Lithium patients and healthy controls was *IL6*. Previous studies on inflammatory changes in mood disorders showed inconsistent results (Sakrajda and Szczepankiewicz 2021). In the study comparing serum levels of inflammation-related cytokines in bipolar patients during manic and depressive episodes, the authors showed a significant increase in IL6 levels during a depressive episode, but in mania, the IL6 level was under the sensitivity range of antibodies, suggesting different, phase-specific cytokine expression patterns in bipolar disorder (Ortiz-Domínguez et al. 2007). However, the study did not include the patients in the euthymic state. Similarly, the recent study presented the relationship between serum levels of inflammatory markers and the severity of bipolar symptoms in the group of drug-naïve BD patients compared to healthy controls (Wu et al. 2023). The authors observed the changes in both pro- and anti-inflammatory cytokine levels in BD (i.a. IL6, IL10 and IL6 /IL10 ratio) during the depression and manic state, suggesting the state-dependent immune dynamic changes. However, the authors did not assess the serum level of inflammatory markers in the euthymic state, the results showed a significant decrease in IL6 level during bipolar depression and severe bipolar mania and decrease in IL10 level during bipolar depression. Contrary to this, the meta-analysis by Rowland et al. (2018) showed that IL6 levels were increased in mania and euthymia patients, but not in bipolar depression compared to the healthy controls. Therefore, it seems that the levels of IL6 in bipolar patients may be phase dependent; however, the exact direction of changes during episodes is still undetermined. The correlation between lithium treatment and IL6 levels also remains inconclusive. In the animal model of mania, the authors showed that lithium administration in rats treated with dextroamphetamine decreased the cytokine level (i.e. IL6) in the frontal cortex, striatum, and serum compared to manic-like rats not treated with lithium (Valvassori et al. 2015). Similarly, the in vivo study on lithium-treated rats presented reduced IL6 levels within the orbitofrontal cortex after lithium treatment (Adams et al. 2020). Contrary results were shown in the in vitro study by Petersein et al. (2015) who investigated the effect of lithium alone, and in combination with antidepressants, or antidepressant alone on cytokine levels in the in vitro study based on healthy subjects’ whole blood assays. The treatment increased levels of IL1B, TNFA, and IL6 in the samples treated with lithium alone and combined treatment with lithium and antidepressants compared to pre-treatment levels. The presented results were observed regardless of immunological pre-treatment stimulation of the blood samples with OKT3/5C3 or phytohemagglutinin, indicating the direct influence of lithium treatment on those cytokine levels. However, that study included only samples from healthy subjects, which may not reflect the cytokine changes in BD patients.

In regard to IL10, our study showed elevated *IL10* expression only in the BD-Lithium group compared to healthy controls but no differences were found between the BD-OD group and controls. IL10 is one of the key mediators of anti-inflammation, regulating the response to pathogens and homeostasis but also regulating basic neural and adipose cell processes (Rutz and Ouyang 2016; Saraiva et al. 2020). The previous studies reported changes in the IL10 protein level and mRNA expression in the peripheral samples from BD patients. Brambilla et al. (2014) showed the decreased expression of *IL10* in blood of BD patients compared to SCZ and healthy controls, thus suggesting the decreased anti-inflammatory M2 signature of peripheral macrophages. Contrary to this, the study including the first-episode BD patients showed an elevated plasma level of IL10 in BD patients compared to healthy controls (Lesh et al. 2018). Interestingly, similar results were obtained in the study, including the clinical staging of BD. Authors presented the increase in IL10 concentration of early-stage euthymic BD patients compared to late-stage, healthy siblings and healthy controls (Tatay-Manteiga et al. 2017). The studies on the first-episode BD patients and early-stage euthymic BD patients may support the stage-dependent increase of IL10, which decreases with the development of the BD, which will explain the heterogeneity of the reported results. The elevated expression of *IL10* observed in the BD-Lithium patients compared to healthy controls, showed in our study, is consistent with previous studies showing that lithium might enhance *IL10* expression (Barbisan et al. 2017). In addition, the in vitro study based on induced immature dendritic cells (iDCs) that compared the valproic acid and lithium treatment during iDC differentiation and maturation showed that both lithium and valproic acid modulate inflammatory response, but only lithium significantly enhanced the production of IL10 before and after the LPS-stimulated DC maturation (Leu et al. 2017). In the context of the above studies, elevated expressions of *IL6* and *IL10* in lithium-treated BD patients showed in our study may suggest the immunomodulatory role of lithium in restoring immunological homeostasis in BD patients. However, further assessment of drug-naïve patients should be performed to discriminate whether the presented changes are results of the mood stabilizer treatment or the course of the disorder.

The main limitation of our study is the relatively small sample of patients, and therefore, future studies on the larger sample size are necessary to better characterize the observed association between *AHI1* haplotype and expression and lithium response as well as treatment dependent changes of immunity-related gene expression. Another limitation is a lack of drug-naïve BD patients group. Involvement of that group in future studies may clarify if changes in gene expression result from bipolar disorder regardless of treatment. The longitudinal approach will allow us to verify the state-dependent expression changes reported in previous studies.

## Conclusion

The observed association of *AHI1* haplotype and the expression in the lithium response in bipolar patients suggest this gene may be involved in the lithium mode of action. Moreover, mood-stabilizing treatment seems to influence the inflammasome and inflammasome-regulated cytokines in euthymic bipolar patients. However, the lack of drug-naïve BD patients did not allow us to determine whether these changes result from mood-stabilizing treatment in BD patients or stem from the disease itself. Although both groups of BD patients presented changes in pro-inflammatory gene expression compared to the control group, only lithium-treated patients showed elevated expression of *IL10*, the anti-inflammatory cytokine, which may support the lithium immunomodulatory properties in bipolar treatment.

## Data Availability

Data is available upon reasonable request to the corresponding author.
